# Comparison of Stool Myeloperoxidase Levels and Their Association With Clinical Parameters in Children With Severe Acute Malnutrition, Children With Moderate Acute Malnutrition, and Healthy Children

**DOI:** 10.7759/cureus.97072

**Published:** 2025-11-17

**Authors:** Charu John, Sunil Kumar Rao, Anil K Saroj, Chandradeep Srivastava, Gopal Nath

**Affiliations:** 1 Pediatrics, Institute of Medical Sciences, Banaras Hindu University, Varanasi, IND; 2 Microbiology, Institute of Medical Sciences, Banaras Hindu University, Varanasi, IND

**Keywords:** fecal biomarker, gut inflammation, moderate acute malnutrition, myeloperoxidase, sever acute malnutrition

## Abstract

Background: Environmental enteropathy (EE) is a sub-clinical inflammatory condition of the intestine that contributes to malabsorption, growth failure, and impaired immunity in children from low-income countries. Stool myeloperoxidase (MPO) is a potential biomarker for assessing gut inflammation in malnourished children. The aim of the study was to estimate the MPO level in children with severe acute malnutrition (SAM), children with moderate acute malnutrition (MAM), and healthy children, and its association with clinical parameters.

Methods: This prospective observational study was conducted from September 2018 to May 2020 in a tertiary care teaching hospital in northern India. Inclusion criteria were children aged 2-60 months admitted/attending the outpatient department with WHO criteria of SAM and MAM, and age and sex matched healthy children. Variables recorded were age, sex, and socioeconomic status, and clinical data, including detailed history, anthropometry, systemic examination, and laboratory investigations. Human MPO enzyme-linked immunosorbent *assay (*ELISA) Kit (ELK1062; ELK Biotechnology Co., Ltd, Wuhan, China) was used for estimating stool MPO levels. The levels were measured according to the manufacturer's guidelines, and the detection level ranged from 1.57 ng/ml to 100 ng/ml. A p-value <0.05 was considered significant. Receiver operating characteristic curve (ROC) analysis was performed to determine the predictive value of MPO for gut inflammation.

Results: A total of 61 SAM, 25 MAM, and 15 healthy children were included. Median MPO levels were significantly higher in SAM (20,000 ng/g) compared to MAM (9,500 ng/g, p=0.004) and healthy children (8,250 ng/g, p<0.001). MPO levels were not significantly associated with diarrhea (p=0.82), edema (p=0.83), or stunting (p=0.86). ROC analysis identified a cut-off MPO level of 6,875 ng/g, yielding 98% sensitivity and 93% specificity for differentiating gut inflammation in SAM versus healthy children with an area under the curve (AUC) of 0.84 (95%CI, 0.77-0.93) (p=0.000).

Conclusion: SAM exhibited significantly elevated MPO levels without clinical correlation, indicating sub-clinical gut inflammation. MPO shows promise as a sensitive biomarker but requires validation in larger, longitudinal, and multi-biomarker studies.

## Introduction

Environmental enteropathy (EE) is a subclinical chronic inflammation of the intestine without overt diarrhea. Persistent fecal-oral exposure to enteropathogens and nonpathogenic fecal microbes leads to immune stimulation or distortion of intestinal microbiota composition in children from low-income countries [1,2].

EE is histologically characterized by small intestine villous blunting, altered barrier integrity, reduced intestinal absorptive capacity, and infiltration of lymphocytes and plasma cells [1]. It contributes to linear growth failure (stunting), subclinical malabsorption of macro and micronutrients, increased gut permeability, poor oral vaccine efficacy, failure of nutritional interventions, growth faltering, and impaired child development [2,3]. The prevalence of EE is underrepresented in the literature due to the lack of non-invasive diagnostic methods and the asymptomatic nature of the condition in many cases.

A critical functional characteristic of EE is altered small intestinal permeability, leading to a measurable increase in flux of small water-soluble molecules across epithelial cells. The lactulose-mannitol test has been widely used as a surrogate for the gold standard for diagnosis, especially when biopsies are difficult to obtain. The prevalence of EE ranges from 29% to 36% when estimated by D-xylose or lactulose-mannitol test, and is higher (57%) on histological examination of the intestine of children with chronic diarrhea [4,5].

Stool myeloperoxidase (MPO) is a potential biomarker for assessing gut inflammation in malnourished children. A multi-site prospective birth cohort study including India, Pakistan, and Bangladesh revealed that MPO levels in the stool of asymptomatic children were five times higher than non-tropical references [6]. The present study estimates the MPO levels in the stools of children with severe acute malnutrition (SAM), children with moderate acute malnutrition (MAM), and healthy children, and assesses their association with clinical parameters.

## Materials and methods

This is a prospective observational study, conducted from September 2018 to May 2020 in the Department of Pediatrics, Institute of Medical Sciences, Banaras Hindu University, Varanasi, India.. The study was approved by the Ethical Committee, Banaras Hindu University Institute of Medical Sciences (approval number: Dean/EC/2020/2019, dated December 7, 2020), and informed consent was obtained from the parents before the study.

Eligibility criteria

Inclusion criteria were children aged 2-60 months admitted/attending the outpatient department (OPD) with weight for height (WHZ) <-3SD and/or mid-upper arm circumference (MUAC) < 11.5 cm and/or nutritional edema [7]. Exclusion criteria were inborn errors of metabolism, HIV positive children, renal tubular acidosis, celiac disease, children with cancer, and children on long-term medication. All children aged 2-60 months admitted/attending OPD were screened for inclusion criteria as per the study protocol, after recruitment of participants.

Sample size estimation

We used the following formula to calculate the sample size: $n = (1.96)^2 \times P(1 - P) / D^2$, where P is the prevalence (0.20) and D is the absolute precision (0.10). Assuming a 20% prevalence (P) of EE, with an absolute precision (D) of 10% and a 95% confidence level (CI), a sample size of 61 SAM was required for this study. Additionally, 25 age-sex matched MAM (WHZ: -2 to -3 SD and MUAC 11.5-12.5 cm) and 15 age-sex matched healthy children were subjected to stool MPO estimation.

Data collection

Variables recorded were age, sex, and socioeconomic status, clinical data, including detailed history, anthropometry (length/height, weight, mid-upper arm circumference), systemic examination, and laboratory investigations. Morning stool samples of children without diarrhea were collected in a sterile container, and for those children who presented with diarrhea, samples were collected after subsiding of loose motions. Collected stool samples were preserved at -80°C in the Departmental Research Laboratory. Human MPO enzyme-linked immunosorbent assay (ELISA) Kit (ELK1062; ELK Biotechnology Co., Ltd., Wuhan, China) was used for measuring stool MPO levels. The levels were measured according to the manufacturer's guidelines, and the detection level range was 1.57-100 ng/ml.

Processing of stool samples and estimation of MPO

Of each stool sample, 100 mg was diluted with 900 µl of phosphate-buffered solution (PBS), and 100 µl of this suspension was added to each well and centrifuged for five minutes. Each centrifuged tube was incubated for 80 minutes at 37 °C three times. Cells in the sample were washed three times in pre-cooled PBS. We added 100 µl biotinylated antibody working solution to each well and incubated at 37 °C for another 50 minutes. We washed the plate three times, to each well we added 100 µl of Streptavidin HRP (horseradish peroxidase) working solution and incubated at 37 °C for 50 minutes.

Again, the plate was washed five times, and 90 µl of ready-to-use tetramethylbenzidine (TBM) substrate solution was added, and this was incubated at 37 °C for 20 minutes. We added 50 µl of stop solution and shook the plate on the plate shaker for one minute to mix. We then immediately recorded the optical density at 450 nm, and MPO concentrations were determined by constructing a standard curve with MPO concentration (ng/ml) on the x-axis and optical density (absorbance) on the y-axis. The final concentration for each sample was obtained by multiplying the measured value by 500 (dilution factor); the product was expressed as MPO ng/g.

Statistical analysis

Data was analysed using SPSS Statistics for Windows, version 16 (SPSS Inc., Chicago, Illinois, United States). The numerical data were represented as mean (SD), median (interquartile range (IQR)), and frequency (percentage). For comparison of two groups, the Student’s T-test was used for parametric data and the Mann-Whitney U-test was used for nonparametric data. A p-value <0.05 was considered significant. Receiver operating characteristic curve (ROC) analysis was performed to determine the predictive value of MPO for gut inflammation.

## Results

A total of 61 SAM, 25 MAM, and 15 healthy children were included. Nearly half of the participants were aged 2-12 months (n=29; 47.5%), and there was a male predominance (n=37; 60.6%). The majority (n=39; 63.9%) belonged to the lower socioeconomic class IV, immunization coverage was seen in 36 (59%), while exclusive and complementary feeding rates were 34.4% (n=21) and 24.5% (n=15), respectively. Common clinical symptoms included loose motion (n=30; 49.1%), edema (n=16; 26.2%), and skin changes (n=38; 62.2%). The baseline characteristics and laboratory parameters of study participants are given in Table 1.

**Table 1:** Baseline characteristics of study cohort (N=61) IQR: interquartile range; SD: standard deviation

Characteristics		Values
Age (months), n (%)	2-12	29 (47.5%)
	13-36	22 (36%)
	37-60	10 (16.4%)
Male sex, n (%)		37 (60.6%)
Weight (kg), mean±SD		6.15±2.27
Height (cm), mean±SD		70.44±11.73
Mid Upper Arm Circumference (cm), mean±SD		10.31±1.6
Socioeconomic Status, n (%)	Class-III	16 (26.2%)
	Class-IV	39 (63.9%)
Immunized, n (%)		36 (59%)
Breastfeeding, n (%)	Exclusive Breastfeeding	21(34.4%)
	Complementary Feeding	15 (24.5%)
Loose Motion, n (%)		30 (49.1%)
Edema, n (%)		16 (26.2%)
Skin Changes, n (%)		38 (62.2%)
Hemoglobin (g/dl), mean±SD		8.9 (2)
Total Leucocyte Count (x10^3^), median (IQR)		13300 (10400, 17800)

Stool MPO levels were significantly elevated in SAM compared to MAM (median: 20,000 ng/g vs. 9,500 ng/g, p=0.004) and SAM compared to healthy children (median: 20,000 ng/g vs. 8,250 ng/g, p<0.001). MPO levels did not show significant variation based on the presence of diarrhea (p=0.82), edema (p=0.83), or stunting (p=0.86). Additionally, there was no significant correlation between MPO levels and TLC (p=0.51) or different age groups (p=0.31) (Table 2).

**Table 2:** Myeloperoxidase levels in the study cohort (N=61) *p-value was calculated by the Mann-Whitney U test. SAM: children with severe acute malnutrition; MAM: children with moderate acute malnutrition; TLC: total leukocyte count

Variables		Myeloperoxidase (ng/g), median (IQR)	p-value*
SAM (n=61)		20000 (13200, 44200)	0.004
MAM (n=25)		9500 (8000, 21200)	
Healthy Children (n=15)		8250 (6125, 9500)	<0.001
Diarrhea	Yes (n=30)	19500 (17500, 34400)	0.82
	No (n=31)	20000 (12200, 55000)	
Edema	Yes (n=16)	18750 (14000, 32400)	0.83
	No (n=45)	20000 (13000, 77500)	
Stunting	Yes (n=47)	20000 (12600, 43500)	0.86
	No (n=14)	19000 (15900, 66200)	
TLC	Normal (n=24)	20800 (17100, 49400)	0.51
	Abnormal (n=37)	19000 (11900, 44200)	
Age interval (months)	1-12 (n=29)	18000 (12600, 50000)	0.31
	13-36 (n=22)	21200 (16500, 34400)	
	37-60 (n=10)	26200 (11900, 100000)	

**Figure 1:**
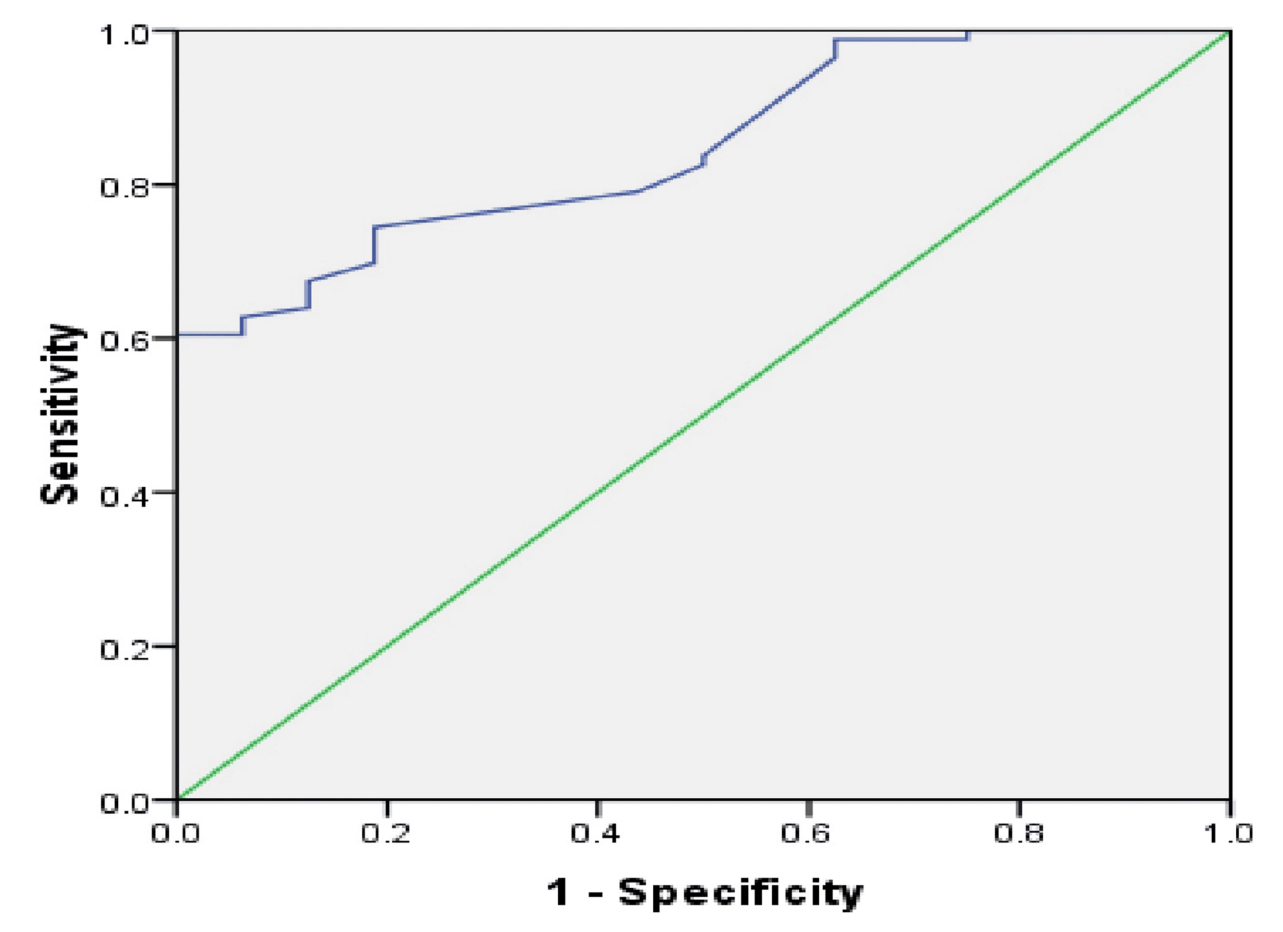
ROC curve for MPO levels predicting gut inflammation in malnourished children An AUC of 0.84 (95% CI: 0.77–0.92, p=0.000) is seen. The optimal cut-off level for MPO was 6875 ng/g stool, yielding 98% sensitivity and 93% specificity in differentiating gut inflammation between malnourished children and healthy children. ROC: receiver operating characteristic; AUC: area under the curve; MPO: myeloperoxidase

The ROC curve for MPO levels predicting gut inflammation in malnourished children showed an area under the curve (AUC) of 0.84 (95%CI, 0.77-0.93) (p=0.000). The optimal cut-off level for MPO was 6875 ng/g, yielding 98% sensitivity and 93% specificity in differentiating gut inflammation between SAM and healthy children (Figure 1).

## Discussion

The optimal value for stool MPO among children under five years of age remains unclear. Studies from high-income countries have used <2000 ng/g as the normal cut-off value [6,8]. The median (IQR) values of MPO in the present study were substantially higher than high-income countries because the case mix of the studied population is different, i.e, children under the age of five in a low and medium-income country, rather than older adolescents and adults suffering from inflammatory bowel disease in high-income countries [6,8]. The median values of MPO levels in the present study were 20000 vs 9500 vs 8250 for SAM, MAM, and healthy controls, all of which were higher than values observed (case vs control, 918.2 vs 812.8 ng/ml) in a study from rural Odisha, which included healthy children from predefined control and intervention villages [8]. This difference might be because of case mixing, as the present study includes SAM and MAM; however, our healthy controls have higher values, which reflect regional and geographical differences of poor sanitation, hygiene, and sources of drinking water.

The median (IQR) value from the Indian sample in the Malnutrition and Enteric Diseases (MAL-ED) study was 14,574.97 (6093.03, 27,507.40) ng/mL for MPO [9], which was lower than the values observed in SAM in the present study (19,500 (17,500, 34,400) ng/g). Whereas MAM and healthy controls in our study have median MPO values lower than values observed in the MAL-ED study [9]. The mixed observation in our study may be because of differences in the targeted population, as the MAL-ED data were collected from infants at three, six, and nine months of age, while our data include children aged 2-60 months. A study of Bangladeshi children in a wider age group of 3-21 months [10] demonstrated lower median values of MPO than observed values in the present study and the MAL-ED study. Prior studies suggest that concentrations of fecal biomarkers may decrease with child age, with levels stabilizing after two years of age [9,11].

MPO is a marker of intestinal inflammation and TH1 immune activation [12]. We showed that the values of MPO were similar in children with or without diarrhea. These observations suggested that chronic gut inflammation persists even in the absence of diarrhea. Similar observations were reported by different authors that intestinal damage does not always present with clinical symptoms but correlates with systemic inflammation and poor outcomes [3,13,14]. While studies in Ethiopia linked elevated MPO levels to poor sanitation, enteric infections, and malabsorption, suggesting regional differences in inflammation severity [15]. A study by Sinhoray et al. confirms that a water and sanitation intervention reduced intestinal permeability but had no significant effect on MPO levels, indicating that gut inflammation persists despite improvements in hygiene [8]. This observation is in line with the hypothesis that sub-clinical gut inflammation can occur independently in children belonging to low and middle-income countries due to persistent fecal-oral exposure to enteropathogens.

The MAL-ED study reported that a composite disease activity score incorporating MPO predicted a loss of 1.08 cm in linear growth over six months [2,8]. Similarly, a study in rural Odisha found an inverse association between MPO levels and height-for-age z-scores (HAZ), confirming that gut inflammation is strongly linked to stunting [8]. Contrary to this, we found no significant associations between MPO levels and stunting (p=0.86). This might be because of a cross-sectional design, which prevents the assessment of changes over time. The present study compared the values of MPO from healthy controls but did not use an independent gold standard for gut inflammation (e.g., intestinal biopsy or validated biomarker panel). Moreover, it suggests only moderate discriminatory ability to predict gut inflammation among children with significantly increased stool MPO levels. 

Limitations of the study

There are some limitations, including it being a single-centre study, the small sample size, and the cross-sectional design, which prevents the assessment of changes over time. We used a single stool biomarker to assess gut inflammation instead of an intestinal biopsy or validated stool biomarker scores. Future studies would need to establish normal values of fecal biomarkers of gut inflammation in Indian children with longitudinal follow-up and from different regional and geographical areas. 

## Conclusions

The present study highlights the high burden of gut inflammation in children with SAM, MAM and healthy children. Ths lack of correlation with symptoms suggests that gut inflammation in malnutrition is largely subclinical and persistent. MPO shows promise as a sensitive biomarker but requires validation in larger, longitudinal, and multi-biomarker studies. 
